# Acknowledgment to the Reviewers of *Metabolites* in 2022

**DOI:** 10.3390/metabo13010121

**Published:** 2023-01-13

**Authors:** Metabolites Editorial Office

**Affiliations:** MDPI AG, St. Alban-Anlage 66, 4052 Basel, Switzerland

High-quality academic publishing is built on rigorous peer review. *Metabolites* was able to uphold its high standards for published papers due to the outstanding efforts of our reviewers. Thanks to the efforts of our reviewers in 2022, the median time to first decision was 13 days and the median time to publication was 34 days. Regardless of whether the articles they examined were ultimately published, the editors would like to express their appreciation and thank the following reviewers for the time and dedication that they have shown *Metabolites*:
A. GanesanLaura CotoiA. Robles-MarhuendaLaura García-CorzoAalim WeljieLaura Giuseppina Di PasquaAarti SharmaLaura MitreaAasim MajeedLaura SantagostiniAbazar GhorbaniLaura SchnackenbergAbdolreza HosseindoustLaura ScranoAbdul BasitLaura StefaniAbdul QayyumLaurel O. SillerudAbdul SirajLaurent MetzingerAbdullah AlnuqaydanLaurentia Nicoleta GaleșAbedalrhman AlkhateebLawrence A. VernettiAbhiram ArunkumarLe XuAbhishek GuptaLea WagmannAbraham Wall MedranoLee Samüel WannAbu Hena Mustafa KamalLee Seong WeiAbubakr H. MossaLeena KadamAbul Kalam AzadLei DengAbuzer AliLei Wang (China)Adam HuczyńskiLei Wang (USA)Adam MatkowskiLeilson BezerraAdam ReichLeng Wei KhooAdam SchrumLenka ŠlachtováAdel PezeshkiLeo K. IwaiAdeline BertolaLeonardo BrunettiAdina SantanaLeonardo Dos Reis SilveiraAdnan IsmaielLeonardo Henrique Dalcheco MessiasAdnan KhanLeonor MeiselAdnan MustafaLeonor Puchades-CarrascoAdolfo Andrade CettoLeszek SzablewskiAdolfo Francesco AttiliLeticia González-MayaAdrian ArendowskiLetizia PalombaAdriána LiptákováLew Lee ChingAdriana MikaLi QiangAdriana TrifanLi WangAdriana VenturaLiana Claudia SalanţăAdriano AraujoLiang LiAdrie VerhoevenLiang Ting LinAfsha TabassumLiang ZhongAftab ShaukatLicio CollavinAgnieszka HerosimczykLídia GonçalvesAgnieszka Owczarczyk-SaczonekLieselot HemeryckAgnieszka PotęgaLihong HaoAgnieszka Wiszniewska-ŁaszczychLilach SoreqAgustin Gonzalez-VicenteLina GaoAgustín Llopis-GonzálezLinda PetijováAhmad AlbaghdadiLindsay BrownAhmad Khusairi AzemiLindsey MillerAhmar RaufLindsy KassAhmed A. ElolimyLinlin GuoAhmed A. ZakiLinxi YuanAhmed AbdellatifLiping LuoAhmed BakillahLiping SunAhmed E. Abdel MoneimLiqing ZangAhmed El GhorabLisbet BrandiAhmed GabrLiu LiuAhmed M. RamadanLiubov GorbachevaAhmed M. SayedLiubov N. SkrypnikAhmed NafisLiudmyla L. TitovaAhmed S. MandourLiudmyla V. GarmanchukAhmed SayedLivleen ShuklaAhmet TopalLizelle PiaterAida MetoLorenzo BacigalupoAidin ForoutanLorenzo DaDaltAike JeuckenLorenzo GuzzettiAin RaalLorenzo Rivas-GarcíaAi-Ru HsiehLory Saveria CrocèAjay KumarLothar BreckerAjay RajaramLouis Kuoping ChaoAjjampura C. VinayakaLuana Beatriz Dos Santos NascimientoAkalesh Kumar VermaLuboslav StárkaAkanksha SharmaLuca CiceroAkilavalli NarasimhanLuca Di GianfrancescoAkio NakashimaLuca GiacomelliAkira GotoLuca Giovanni CampanaAkira HondaLuca GoldoniAkira SugawaraLuca LaghiÁkos KenézLuca MoriniAlain PerretLuca PasquiniAlan WongLuca ScottiAlbert KoulmanLucas Freitas De FreitasAlberto BartoloméLucia BailoniAlberto ChighineLucia La SalaAlberto Jorge Oliveira LopesLucía Méndez LópezAlberto Rodríguez-ArchillaLucia StančiakováAlberto SalomoneLucian CopoloviciAlberto SpisniLuciane Viater TureckAlbino MaggioLucio Della GuardiaAldo TavaLucjusz ZaprutkoAldona KasprzakLudmila Danilowicz-SzymanowiczAlejandro A. TapiaLudmila KazdovaAlejandro CáceresLudovina GalegoAlejandro Castro-AlvarezLuigi MandrichAlejandro LaraLuigi MilellaAlejandro PlascenciaLuis Angel Medina JuarezAlejandro SanzLuís C.N. Da SilvaAleksandra CzumajLuís CardosoAleksandra JoticLuis Cláudio Nascimento Da SilvaAleksandra KopaczLuís CrisóstomoAleksandra KuzanLuis EspinozaAleksandra MišanLuis GoyaAleksandra RanitovićLuis O. Soto-RojasAleksandra S. KristoLuis RodrigoAleksandra SentkowskaLuis Sarmiento-FrancoAlena PechovaLuis VarelaAleš BlincLuiz Leonardo SaldanhaAleš JerinLuiz Palucci-VieiraAlessandra FerramoscaLukáš NajdekrAlessandra PalladiniLukasz JaremkoAlessandra TataŁukasz M. MilanowskiAlessandro BenedettoŁukasz MyczkoAlessandro BonardiŁukasz SzeleszczukAlessandro De SireŁukasz SzymańskiAlessandro DelitalaLuminita MoraruAlessandro FratiLuo-Yang DingAlessandro GambellaLu-Sheng HsiehAlessandro GuerriniLuz Maria Del Razo JiménezAlessandro MalobertiLydie Nadal-DesbaratsAlessandro MeduriLynnette M. JonesAlessandro PasseraLyubov E. SalnikovaAlessia VignoliLyudmila Bel'skayaAlex Buoite StellaLyudmila RachekAlex RafachoLyudmila V. YansholeAlexander A. GulinM. Ariel WallaceAlexander Benedikt LeichtleM. Dulce EstêvãoAlexander BerezinM. Eduviges Burrola-BarrazaAlexander Da Silva ValeM. JameelAlexander J. KnightsM.R. KabilovAlexander L. SemenovMaciej ChęcińskiAlexander V. OleskinMaciej JarzebskiAlexander ZhgunMaciej TarnowskiAlexandr KokovMaciej UgorskiAlexandra M. GouveiaMadhumita BarooahAlexandra NunesMagdalega BiesagaAlexandra Peregrina LavínMagdalena BartnikAlexandre Lopes EvangelistaMagdalena CalAlexandru PopaMagdalena HurkaczAlexei ChukhlovinMagdalena Krajewska-WłodarczykAlexey GurevichMagdalena Orczyk-PawiłowiczAlexios A. AlexopoulosMagdalena Polak-ŚliwińskaAlexis StamatikosMagdalena WójciakAlfredo CiccodicolaMagdalena Wójciak-KosiorAlfredo G. CasanovaMaha AlhussainAlfredo MiccheliMahbubul MatinAli BoolaniMahdieh AlipourAli Ehsan SifatMahesh AttimaradAli Kerim YılmazMahesh Raj NepalAli SaeedMahipal Singh KesawatAli Salehzadeh-YazdiMahmoud AbulmeatyAlice BonomiMahmoud BalbaaAlicia EstevezMahmoud ElfakyAlicia Mayeuf-LouchartMahmoud ElsayedAlida Kindt-DunjkoMahmoud KandeelAlin CiobicaMahmoud S.M. MohamedAlina Delia PopaMahshid NaghashpourAlina PlenisMailo VirtoAlina PykaMaitree SuttajitAline T. BruniMajdy IdreesAlireza PirzadMajid Sharifi RadAlison G. PaquetteMakoto HaradaAlison J. ForheadMakoto TsunodaAlphus D. WilsonMalbert Charles-HenriAlpo VuorioMałgorzata GodalaAltaira D. DearbornMałgorzata GrabarczykÁlvaro Fernández OchoaMalgorzata GrzesiakÁlvaro José Santos-NetoMałgorzata JeleńAlvaro PérezMałgorzata Kalemba-DrożdżAmanda Almeida De OliveiraMałgorzata Kotula-BalakAmandeep SandhuMałgorzata StachońAmarendra N. MisraMałgorzata SzczukoAmedeo LonardoMałgorzata Szultka-MłyńskaAmel TaibiMalgorzata Trofimiuk-MüldnerAmelia V. García-LuengoMalgorzata WasniewskaAmine ElbouzidiMalgorzata Witkowska-ZimnyAminu MohammedMałgorzata WrzosekAmir AshoorzadehMalgorzata Zakłos-SzydaAmir HadiMałgorzata ZiarnoAmir Mohammad MalvandiManabu NatsumedaAmirata Saei DibavarManik SorenAmit KumarManisha NigamAmitava RakshitManju Rawat SinghAmjad HussainManohar KodavatiAmmad Ahmad FarooqiManoj GhasteAmmar AltemimiManoj KhadkaAmmar BaderManoj Kumar TripathiAmmar TahirManuel Vilares-FerroAmnart PoapolathepManuela GrimaldiAmrinder SinghManuela PeukertAmritlal MandalManzoor RatherAmro AmaraMara CarsoteAmutha RamadasMara MazzoniAmy CunninghamMarc MarescaAmy HarmsMarc-André DelsucAna BratuMarcello CasertanoAna Carolina MoreiraMarcelo CoertjensAna Henriques MotaMarcelo FerreiraAna I. SoteloMarcia FrancoAna Isabel Rocha FaustinoMarcin CzopAna KaicMarcin SiwekAna Luisa PereiraMarcin WłodarczykAna María Calderón De La BarcaMárcio José De Abreu RodriguesAna MornarMárcio RodriguesAna ReisMarco BiagiAna Roca-FernándezMarco CicconeAnamaria SavuMarco CosentinoAnamika PandeyMarco DacremaAnas M. Abdel RahmanMarco Di GioiaAnas ShamsiMarco FioreAnastasios E. GermenisMarco FoganteAnastasios SerbisMarco G. AlvesAnastasiya SolovievaMarco La VerdeAnatoly P. SobolevMarco Matteo CicconeAnca Adriana ArbuneMarco PortelliAnca DumbravaMarco RosinaAnđelija MalenovićMarco RoversoAnders MalmendalMarco SegattoAnders PedersenMarco ZuinAndleeb KhanMarconi Batista TeixeiraAndrás BikovMarcos Arturo Martínez-BanaclochaAndré Alves DiasMarcos BrioschiAndre LechelMarek CzosnykaAndré Luís MoreìraMarek PieszkaAndrea BragaglioMarek SkrzypskiAndrea FerenczMarek WesołowskiAndrea GažováMarek WiergowskiAndrea GelemanovicMargaret GradovaAndrea Gonzalez-MontoroMargarida F. B. SilvaAndrea Herrero-CerveraMargherita CaroliAndrea HuwilerMargherita EufemiAndrea Luísa Fernandes AfonsoMargherita NeriAndrea RagusaMargherita RuoppoloAndrea RomaniMaría Dolores Ortiz-MasiáAndrea StoccoroMaria Aurora MoralesAndrea TittarelliMaria BogdanAndrea VecchiolaMaria BucovaAndreadi AikateriniMaria Consuelo MuraAndreas G. LösslMaria D. Giron-GonzalezAndrei Gheorghe MotocMaria Da Graça Costa MiguelAndrei I. KhlebnikovMaria De La Luz Garcia PardoAndrei Vasile NastutaMaria De MieriAndreia GarcêsMaria Do Carmo PeluzioAndreia RodriguesMaria Elena PisanuAndrej KovacMaría Elvira López-OlivaAndreja JakasMaria Enrica Di PietroAndres Barajas-SolanoMaria Fernanda Hornos CarneiroAndrew J. ChetwyndMaria FortofoiuAndrew J. RosendaleMaria Giovanna BuonomennaAndrey ChibiryaevMaria Grazia CappaiAndrey Poddel'skyMaria Grazia Di CertoAndris JankevicsMaría Isabel Rodríguez LópezAndrzej SzutowiczMaria JenmalmAndy ChetwyndMaría Jesús OrtegaAneliya MilanovaMaria João RamalhosaAneta NowakiewiczMaría José García-PolaAneta Ostróżka-CieślikMaria KomelkovaAng LiMaria Luisa DettoriAngel Josabad Alonso-CastroMaria Luisa GarrèÁngel LlamasMaria Luisa MalosioAngela BachiMaria Luiza S. MelloAngela BolzonelloMaría Luz CouceAngela OttoMaría M. Morales Suárez-VarelaAngela PapaMaria Malvina TsamouriAngela StaicuMaria Margarida MateusAngela-Patricia HernándezMaria Michela SalvatoreAngélica Román GuerreroMaria Morgan-BathkeAngeliki AngelidiMaria N. TutukinaAngeliki KatsafadouMaria NotarnicolaAngelo GismondiMaría Orosia Lucha-LópezAngelo M CarellaMaria P. RubtsovaAngelo TorrenteMaría Rocío Rodríguez ArcosAngelos SikalidisMaria SokolAnh NguyenMaría-Jesús Tabernero-DuqueAnibh M. DasMaría-José ArgenteAnika KükenMarialuisa MennaAnil SinghMariam GaidAnil TharappelMariana FloriaAnila IqbalMariana MaronezeAnis Ben HsounaMariana PalmaAnja Kathrin JaehneMariana-Atena PoianaAnja SipkaMariann HarangiAnjali A. SatoskarMarianna MazzaAnjas A. SamsudinMariano González-PérezAnju KumariMarie Claude MenetAnkit RochaniMarie SjögrenAnkit ShroffMarie-Joelle VirolleAnkita PunethaMariia NesterkinaAnna ArtatiMarijana SkorićAnna BirkováMarijana Zovko KončićAnna CieślińskaMarika CordaroAnna CzarneckaMarina CaldaraAnna HalamaMarina PiscopoAnna JakubczykMarina V. PadkinaAnna Jakubska-BusseMarine M. LetertreAnna Kablak-ZiembickaMarino VilovićAnna LipertMario OlleroAnna RysiakMario SimirgiotisAnna SansoneMario SoaresAnna SikoraMario Ulises Pérez-ZepedaAnna StachyraMario Vincenzo RussoAnna StefanskaMariola BochniarzAnna Szczerba-TurekMarion DalmassoAnna Tabęcka-ŁonczyńskaMarius CrainaAnnalisa ChiavaroliMarius Emil RusuAnnarita NappiMarius MihasanAnne AndersonMariusz SikoraAnnie JoubertMariusz Tomasz DziadasAnnunziata NuscaMark SterkenAnoop KumarMark TarnopolskyAnoosh RakhshandehMarkandan ManickavasagamAnthony QuéroMarko MarhlAntje Büttner-TeleagăMarko NovakovićAntoaneta TrendafilovaMarko R. CincovićAntoine CherixMarkus M. HoffmannAnton KiselevMarkus NaglAnton KováčikMarlene Fabiola Escobedo MongeAntonella Caterina BocciaMarta CarettoAntonella TinelliMarta KarazniewiczAntonello SantiniMarta KaszowskaAntoni SzumnyMarta KordalewskaAntonija TadinMarta KrasnyAntonina ArgoMarta OpalińskaAntonino LauriaMarta PotrykusAntonio BusquetsMarta R. RomeroAntonio FacciorussoMarta TomczykAntónio FerreiraMarta Woźniak-KarczewskaAntonio Francesco Maria GiulianoMartin BartkovskýAntonio GiordanoMartin Christian MichelAntonio Giovanni SolimandoMartin E. BlohmAntonio GiovinoMartín González-AndradeAntonio González-BulnesMartin KollerAntonio LucacchiniMartin LeníčekAntonio ManentiMartin M. MortazaviAntónio NogueiraMartin PéčAntonio OrlacchioMartin RasporAntonio Ramos-MartinezMartin SvobodaAntonio RandazzoMartin TeraaAntonio Sarasa-CabezueloMartina CrociatiAntonio Victor Campos CoelhoMartina MaggiAnup S. PathaniaMartina MiluchováAnurag PassiMartina Zandl-LangAnusorn CherdthongMartis GeorgianaArancha Llama-PalaciosMartyna BatorskaArash Zarrine-AfsarMartyna WilkAravind JukantiMaruti Jayram DhanavadeArcady MarkelMarvin A. Soriano-UrsúaArcady PutilovMarwa A.A. FayedArda YıldırımMary BoardAri Satia NugrahaMary C. MajAriane RozzaMaryam GoudarziArijita SarkarMaryna De WitArinc OzturkMasafumi NodaAristidis TsatsakisMasahiro SugimotoArjun H. BanskotaMasamitsu MaekawaArjun SapkotaMasanori AritaArjun SenguptaMasanori FukushimaArmando ZarrelliMasayuki NagasawaArmandodoriano BiancoMasayuki SatakeArnab MajumdarMasayuki TanabeArne UlbrichMasayuki YamamotoArnulfo Ramos-JiménezMasoume AmirkhaniArtem RogachevMassimiliano RuscicaArthit PhosriMassimo CapocciaArtur BanachMassimo IacovielloArtur KrzyżakMateja GermArturo LópezMateus Pies GionbelliAruliah RajasekarMateusz WoźniakArzu UçarMathieu LaplanteAsada LeelahavanichkulMati PääsukeAshenafi F. BeyiMatias Monsalves-AlvarezAshkan ZandiMatilla-Escalante Diana C.Ashley J. SniderMatthew PaulAshot SargsyanMattia ChiesaAshu JohriMaurício Laterça MartinsAshutosh ManiMaurizio BadianiAsim Kumar BepariMaurizio MargaglioneAsli YilmazMaurizio PaciAsmaa KhafagaMauro ColucciaAswatha Ram H.nMauro FinicelliAswin VerhoevenMauro LombardoAtanu PatiMax PiffouxAtique Ahmed BehanMaximilian KleinertAtish MukherjiMaximilian ZeydaAurel Popa-WagnerMaya ZachutAurelio San-MartínMayank ChoubeyAutumn MarshallMayke OosterlooAvinash Chandra PandeyMayo TsuboiAxel SchmidtMayur DokeAyesha TahirMazen AlmehmadiAyman MahmoudMazen NazalAysel OzpinarMazen SalehAyush RanawadeMazhar HussainAzadeh AsefnejadMd Khalid AnwerAzzurra StefanucciMd Nazmul Hasan ZilaniBabett RamsauerMd. Moshfekus Saleh-E-InBal Krishna ChaubeMedha PriyadarshiniBala Krishna PrabhalaMedine Cumhur CüreBalamuralikrishnan BalasubramanianMeena Kishore SakharkarBalu Alagar Venmathi MaranMegan L. FalsettaBang-gee HsuMegan MarronBaobei WangMehmet BozkurtBarbara Ruszkowska-CiastekMehmet SarierBarbara SawickaMehran AkselBarış Ethem SüzekMeliha EkinciBarlin O. OlivaresMengquan YangBart De GeestMian HuangBartosz KruszewskiMichael AzainBaskar VenkidasamyMichael BoschmannBeata Hukowska-SzematowiczMichael H. KogutBeata NaumczukMichael KaestnerBeata Sarecka-HujarMichael KiebishBeatriz I. Fernández-GilMichael LiebmanBeatriz Sabater-MuñozMichael StoskopfBee Kang TanMichael WeichhausBehnam Asgari LajayerMichael WiggsBehzad KavianiMichail KastelloriziosBelém Sampaio-MarquesMichał BiernackiBelén Callejón LeblicMichał KaliszanBen De Lacy CostelloMichał KalitaBenjamin D. BrooksMichal LetekBenjamín PinedaMichal NiemczakBenny BjörkblomMichal NowickiBerenice PalaciosMichal ZmijewskiBernardo BaldisserottoMichela FerrucciBernd DiehlMichela GiulianoBernd StratmannMichele CiullaBetina Cecilia AgarasMichele CostanzoBetty Yuen-Kwan LawMichele MaffiaBhagavathi SundaramMichiel ThomeerBhakti BasuMichinari HiedaBhanwar Lal PuniyaMiguel A. Aguilar-GonzálezBhaswati BhattacharyaMiguel A. OrtegaBhawik Kumar JainMiguel Ángel Hurtado OlivaBiagio BaroneMiguel Ángel PrietoBiagio RaponeMiguel Ramón Pecci LloretBiliana Lozanoska-OchserMihai Dorin VartolomeiBiliana NikolovaMihai OlteanBiljana M. BojovićMihail MitovBimal GhimireMihalj PošaBimal Kumar GhimireMi-Hyun NamBimrew AsmareMike WilliamsonBin HuMikhail Yu GolovkoBin WuMikio HayashiBirgit Lichtenberg-KraagMikkel BrentBoban AndjelkovicMikko Uusi-OukariBoel De PaepeMiklós MézesBogumil BryckiMikołaj GralakBogumiła PilarczykMilan JakubekBojana DanilovicMilena VukicBojana VidovicMilica PešićBojjibabu ChidipiMilka MilevaBoleslaw KarwowskiMilos PjanicBolin ZhangMine KöktürkBonaccorsi Di Patti Maria CarmelaMinerva Codruta BadescuBorislav AngelovMing ChenBoyan GrigorovMing Hsien ChanBoyong KimMing YangBožidar BenkoMingchih FangBrandon L. PearsonMing-Jui HungBrandt BertrandMing-Kuei ShihBrankica FilipicMingxing LeiBratko FilipičMingxun WangBreda Jakovac-StrajnMinjoo KimBrendan M. DugganMin-Sik KimBrian AckleyMioara BercuBrindusa TiperciucMiran ŠebeštjenBruna LarattaMircea Adrian OroianBrunella CapaldoMircea TampaBruno K. RobbsMiren AltunaBruno Leite SampaioMiriama SimunkovaBumjo OhMirjana JerkicByungju LeeMiroslav RadenkovicC. Michael GreenliefMiroslava NedyalkovaC. SanthoshMiroslava RakocevicCagakan OzbalciMiroslaw ZagajaCamelia Elena LuchianMirosława Ferens-SieczkowskaCarla PeregoMiruna NemeczCarlos A.M. CarvalhoMirza PojskićCarlos Eurico Dos Santos FernandesMisbahuddin M. RafeeqCarlos GuardiaMohamad Hesam ShahrajabianCarlos Miguel Henriques FerreiraMohamad Nurul IslamCarlos Pérez-MonterMohamed A. El-EsawiCarlos Sandoval-CastroMohamed Ait-El-MokhtarCarlos Torres-TorresMohamed Al-FatimiCarmela Dell'AversanaMohamed Ali BenabderrahimCarmela SantangeloMohamed E. Abd El-HackCarmelo CorsaroMohamed ElmonemCarmen BediaMohamed F. M. IbrahimCarmen Lidia ChitescuMohamed FodaCarmen Ortega-santosMohamed G. ShehataCarmen PanaitescuMohamed HassanCarmine FinelliMohamed Jawed AhsanCarolina FontanaMohamed Lotfy AshourCarolina OteroMohamed MediouniCaroline UmMohamed OrabiCarolyn A. EcelbargerMohamed SolimanCassandra E. HolbertMohamed W. AttwaCatarina SeabraMohamed Z.M. SalemCatarina SilvaMohammad Abdul Momin SiddiqueCaterina SagnelliMohammad H. SemreenCatherine Cioffi CohenMohammad KhalidCecília De Souza ValenteMohammad QneibiCecilia SamieriMohammad Usman AhmadCedric ChaverouxMohammad Zahirul IslamCengiz TürkseverMohammadreza RezaeigolestaniCerasela Elena GîrdMohammed ElmetwallyChaisak ChansriniyomMohammed Fouad El BasuiniChaiyapas ThamrongyoswittayakulMohammed Oday EzzatChaiyavat ChaiyasutMohan LalChanchal Sur ChowdhuryMohannad IsmailChandrakala Aluganti NarasimhuluMohd Abul KalamChandrashekhar HonraoMohd Helmy MokhtarChang LiaoMohd Nazam AnsariChang-Hoon ChoiMohsin KhurshidChangseon RyuMohtadin HashemiChangwon JeongMoisés Martínez VelázquezChao WuMona El-AasrChao ZhangMonica GalloCharalambos FotakisMonica Guarino AmatoCharalampos SiristatidisMonica Isabella CutrignelliCharat ThongprayoonMónica SantosCharis LiapiMonika BarbaricCharlotte SteenblockMonika GrabiaCharng-Cherng ChyauMonika KosmalaChee Kong YapMonika KoziełCheng WangMonika Marcinkowska-LesiakChengdong HuangMoshiul Islam IslamCheng-Yang HuangMozaniel OliveiraChengyi TuMrigya BabutaChengyue WuMuawuz IjazChenxi CaiMudassar Nawaz KhanCheryl C.H. YangMuhammad A. AlsherbinyChetna SoniMuhammad AfzaalChi Chun WongMuhammad AhsanChi ZhaoMuhammad Dawood ShahChia Shan WuMuhammad Imran TousifChia-che ChangMuhammad Irfan Ur Rehman KhanChia-Chi LiuMuhammad N ZahidChia-Chun TsengMuhammad SohailChiara Maria MottaMuhammad Usama IslamChiara RossoMuhammad UsmanChia-Wei ChengMuhammed KüpeChien-Hsieh ChiangMuhammed Shafeekh MuyyarikkandyChien-Hung LeeMunawar NaylaChih-Ming LinMurali DamaChih-Po ChiangMurat YigitChih-Yang WangMurtaza TambuwalaChinedu Prosper AnokwuruMustafa CesurChing-Jin ChangMustapha Cherkaoui MalkiChing-Yi ChenMuthu PeriasamyChinna Venkata Subbaiah KadiamMuthukumar KannanChi-Rei WuMuthuramalingam PandiyanChi-Ting HorngMuttalip GündoğduChi-Yu LuMya Myat Ngwe TunCho-Hao LeeNaceur MhamdiChris CramerNada M. MostafaChrissoula VoidarouNada SallamChristel NeutNadeem UllahChristian BarbatoNagasuryaprasad KotikalapudiChristian HobsonNagendra ChauhanChristian JungnickelNagwa Ali SabriChristian KosanNaim Abu FrehaChristian U. OeingNallely Rivero-PerezChristie PanissiNana Y. AnkrahChristina CoughlanNanna Hjort VidkjærChristina E. KostaraNaoharu YagiChristine Grosse-BrinkhausNaohisa ShobakoChristoph CastellaniNaoki TanakaChristoph ReinhardtNaoyuki OtaniChristopher GaffneyNarakorn KhunweeraphongChristopher GernerNarayanankutty ArunaksharanChristopher L. CoeNaresh Kumar RajendranChristos K. KontosNarumol JariyasopitChristos PapaneophytouNatalia DrabińskaChuanhai CaoNatalia GrubaChun GaoNatalia GuevaraChun Hung ChuNatalia Ivanovna AgalakovaChung Yeng LooiNatalia KurhalukChung-Jung LiuNatalia ParoulChun-Kuang ShihNatalia SimionescuChunling HuangNatalia SkiepkoChunzhang YangNathan LanningChyntia JasirwanNavaz KharazianCiavoi GabrielaNavid Jubaer AyonCícero CenaNawaf Al-MaharikCicone FrancescoNehaben A. GujaratiCinzia AntognelliNeil ClarkeCinzia BoccaNeil DanielsonClaire StockerNeith PachecoClara ChoNemesio Villa-RuanoClaudia DelgadilloNesrein M. HashemClaudia GenoveseNetta CohenCláudia Jacques LagranhaNeusa L.L. FigueiredoClaudia MartiniNgbolua Koto-te-NyiwaClaudia Muhle-GollNguyen Phuoc LongClaudio BernardazziNguyen Thanh LeClaudio De LazzariNick KouttabClaudio FerranteNico UeberschaarClaudio LuchinatNicola A. UccellaClaudio ParraNicola Brunetti-PierriClaudiu MorgovanNicola CarettaCleverton De AndradeNicolae GicaClinton BruceNicolai PanikovConcettina La MottaNicolás PedriniCong KongNicoleta RaduCongjun LiNidal JaradatCongshan SunNiels Damkjær OlesenConsolato SergiNikhil R. GandasiConstantinos G. TsiafoulisNikiforos AlygizakisConstantinos K. ZacharisNikola ČobanovićCorinne LionneNikolaos A. ChrysanthakopoulosCorrado RomanoNikolaos LabrouCosmas NathanailidesNikolaus GaßlerCosmin CituNikolay Ul'yanovskiyCostanza JuckerNilgün YıldırımCovadonga Lucas-TorresNils Petter KjosCrina Carmen MureșanNing ChenCristian MunteanuNirajan ShresthaCristian ScheauNiranjan ParajuliCristian StătescuNirit BernsteinCristiana Iulia DumitrescuNirupam BiswasCristiane Villela-NogueiraNishida TakehiroCristina AchimNishikant WaseCristina Juan GarciaNobuyuki IshibashiCristina SoaresNobuyuki OkahashiCristina-Mihaela LăcătușuNoemi PolgarCrystell Guadalupe Guzmán PriegoNoha F. AbdelkaderCsaba VáradiNorbert TripoltCuma MertogluNorio YamamotoCunchuan WangNorma OviedoCurtis R. CoughlinNorman RatcliffeCynthia BarreraNorrie PearceCynthia BlantonNoureddine BencheikhD. Mohd. Azam AnsariNoureddine El AouadDae Eun ChoiNunzia IaccarinoDae Yong YiNunziatina De TommasiDagmar PavlůNupur DasDaichi SumiNur Hanisah AzmiDaisuke KobayashiNuray AcarDamiano D'ArdesOana SandulescuDan JiangOana-Maria BolduraDana Carmen ZahaOctavian Tudorel OlaruDana StoianOksana SorokinaDanail PavlovOlaf LarsenDangdang LiOlaia Martinez IglesiasDaniel C. MorrisOleg ShuvalovDaniel JancuraOlfa ZarroukDaniel LiedtkeOlga Czerwińska-LedwigDaniel López MaloOlga DedaDaniel Lozada RamirezOlga I. YarovayaDaniel MauvoisinOlga Martyna Koper-LenkiewiczDaniel MierlitaOlga ZaborinaDaniel NeureiterOlha MykhailenkoDaniel P. PotaczekOlimpio MonteroDaniel Reis WaisbergOliver TuševskiDaniel SmržOliver WeingärtnerDaniel TurudićOlivier LidoveDaniel W. NixonOmkar B. IjareDaniela CaccamoOrnella CapellariDaniela GabbiaOrnella CominettiDaniela HanganuÓscar Lorenzo GonzálezDaniela RagoOscar NunezDaniela RovitoÓscar Rapado-GonzálezDaniela Valdez-JassoOsmel La-Llave-LeonDaniele BottaiOthmane MerahDaniele GarcovichOumaima AmmarDaniele NovielloOzge SahinDaniele VergaraOzohanics OliverDaniil OlennikovPablo Jesús Marín-GarcíaDanijela Ristić-MedićPablo VelascoDanuta GajewskaPalanisamy NallasamyDaphne BazopoulouPalaniyandi KaruppaiyaDaphne T. LianouPalok AichDaquing PiaoPanagiotis KaranisDarin E. JonesPaniz JasbiDar-In TaiPanteleimon G. TakisDarinka AckovaPaola BruniDario SiniscalcoPaola Di PietroDariusz JedrejekPaola QuaresimaDariusz KokoszyńskiPaola RoggeroDariusz KruszkaPaola TuranoDariusz WaniczekPaolo AbondioDarko PreinerPaolo ArosioDasheng LuPaolo CampioniDavid Cole StevensPaolo Emidio CrisiDavid FellPaolo FagoneDavid GaulPaolo MeneguzzoDavid LeanPaolo PolidoriDavid ObesoPaolo TrucilloDavid P. SmithParamita ChakrabortyDavid RondeauParamjit TappiaDavid ŠilhaParaskev Todorov NedialkovDavid Varillas DelgadoParbeen SinghDavid ZweikerParisa GazeraniDavide BarrecaPaschalis-Thomas DouliasDavor PlavecPasquale ParibelloDeanna HorvathPatric DelhantyDebdeep DuttaPatricia Seoane-CollazoDecheng RenPatrick DansetteDeepak KumarPatrick PoucheretDeepanwita BanerjeePatrizia AgrettiDeepika AwasthiPatrizio MazzoneDejan GođevacPatrycja KleczkowskaDelia TitPau Loke ShowDeliang ZhangPaul CarlsonDelicia Shu Qin OoiPaul F. WilliamsDelphine ParrotPaul G. ThomesDemetrios ArvanitisPaul ValleDenisa MarginǎPaula BoaventuraDer-Yuan ChenPaulina KrawiecDhananjay YadavPaulo Ferrez Collett-SolbergDi WuPaulo Riceli RibeiroDiana Andreia Tavares PintoPavan KumarDiana Castro-RodríguezPawel Antoni KolodziejskiDiana Cláudia PintoPawel K. LorkiewiczDiana M. LindquistPaweł KafarskiDiego MoriconiPaweł OlczykDieter RiethmacherPaweł RamosDietmar EnkoPayam BehzadiDiletta Francesca SquarzantiPayam EmamiDimitra Rafailia BakaloudiPedro Ferreira-SantosDimitrios PapadimitriouPedro Martínez-GómezDimitris KardassisPedro Paulo Menezes ScariotDinesh BarupalPedro Vicente MauriDinesh UpadhyaPeggy SekulaDinithi PeirisPeishen (Elva) ZhaoDipankar RoyPekka Keski-RahkonenDipayan BosePeng GaoDivesh SardanaPernille HolmagerDmitriy V MazurovPero MijićDmitry FrankPerumal VivekanandhanDmitry KarpovPéter Ábrányi-BaloghDmitry S. KosyakovPeter ClementsDoaa IbrahimPeter D. KarpDomenico AlbanoPeter HollanderDomenico CucinottaPeter J. OefnerDomenico LioPeter JarcuskaDomenico PecoraPeter MwangiDominic D'AgostinoPeter PensonDong HanPeter PikoDong LiuPeter PolčicDongchul KangPéter PoórDongdong WangPetr ŠimekDong-Kyu KimPetra Lindholm-LehtoDong-Wook HanPetra M. KlingeDonovan McGrowderPetra WolfDorien M. KimenaiPetros DinasDorota WojtysiakPetrova BoryanaDragana Samardžija NenadovPhil ChilibeckDragana ŠunjkaPhil. WhitfieldDuangjai TungmunnithumPhila RaharivelomananaDuncan ToplissPhilip LangeDung Ha-TranPhilip W. WertzDunja SamecPhilippe B. WilsonDurga Kalyani PaturiPia Lopez-JornetDyana OdehPier Paolo GiovanniniE.A SmirnovaPiergiorgio FranciaEberhard UhlPierre-Aurélien BeuriatEbrahim Abbasi OshaghiPietro LombardiEbrahim NorouziPietro TedescoEdgar RuedaPing DongEdith AranyPing-Hsun WuEdson SilvaPiotr DąbrowskiEduard BardajíPiotr DobrowolskiEduardo Molina-JijonPiotr DuchnowskiEduardo Perez-CamposPiotr DziegielEdward EisensteinPiotr F.J. LipińskiEdward KrzyżakPiotr HeczkoEdward N. HarrisPiotr KuczeraEdward QuadrosPiotr StefanowiczEdwin D. LephartPiotr SugierEdyta MolikPolyxeni MantzouratouEdyta SuligaPoonam RanaEider Pascual-CorralesPorfirio NavaEiichi SatoPradeep DewapriyaEkaterina BaranovaPradeep DwivediEkaterina DiachkovaPradeep Kumar SudheeranEl Hadji M. DioumPradoldej SompolEl Hassan SakarPragneya DemeElena D. BazhanovaPramod R. SomvanshiElena De FelicePranothi MulintiElena DintePrasad ParchuriElena GirichPrashant KaushikElena LevantiniPrashant NighotElena ObradorPrashant SinghElena V. TretyakovaPrashanth SuravajhalaElena VillacrésPratibha PandeyEleni AndreouPratik AgrawalEleni BairaktariPratip BhattacharyaEleni KoniariPraveen KogantiEleni RebelosPrawej AnsariEleni T. BairaktariPriyankar DeyEleni TsalikiPrzemysław DudaEleonora CavallariPrzemysŁaw ł. ZalewskiEleonora LaiPushpa Raj JoshiEleonora NapoliQian DuEleonóra SpekkerQiang HuangEleonore FröhlichQiang WangElettra BarberisQiantao ZhengElisa Miriam Valenzuela SotoQikui WuElisabeth PinartQinghua CaoElisabetta CanettaQing-li ShangElisabetta CasalinoQingsen ShangElisabetta GiudiceQingye ZhangElisabetta TostiQingyu ZhouElizabeth FernandesQuan ZhangElizabeth L. HarveyQuanwei ZhangElizabeth NeumannQunzhou ZhangElizabeth R. LusczekRabia Sannam KhanElke AlbrechtRachele NieriEllen Cristini FreitasRaciel Javier Estrada-LeónEllen HertzmarkRadojica DjokovicElmira JalilianRadu AlbulescuElsayed MehanaRadu RacovitaElżbieta CiporaRadu TamaianElżbieta Hać-SzymańczukRafael BruschweilerElżbieta Wałajtys-RodeRafael Coutinho Mello MachadoElżbieta WołejkoRafael Garrett Da CostaEmami Bistgani ZohrehRafael Salto-GonzalezEman E. MahmoudRafał FrańskiEmanuel Hernández NúñezRaffaela PeroEmanuel Hernández-NúñezRaffaele SerraEmanuela ZanardiRaffaella CascellaEmiel Van Der VorstRaffaella CoccoEmil BulatovRaghavendhar R. KothaEmilia VassilopoulouRaghbendra Kumar DuttaEmma D'IppolitoRahila HafeezEmma EvergrenRahul SuryawanshiEmmanuelle BichonRaimundo Fernandes De Araújo JúniorEmoke PallRaja GanesanEng-Keng SeowRajendran SelvakesavanEngy ElekhnawyRajesh ParsanathanEnquan XuRajiv JanardhananEnrico FioreRakesh Kumar BachhetiEnrico SangiovanniRalf BlankEnrique GomezRalf-Erik WellingerEphrem EngidaworkRam Babu UndiEric ChappelRamesh Kumar SainiEric De GrootRamgopal KashyapEric DeslandesRamon I. SantamariaEric Francelino AndradeRamon LavadoEric PiverRamos PatricioEric YagerRana Muhammad AadilErick Martínez HerreraRandal S. StahlErik BehringerRandolph D. GlickmanErika CioneRani PallaviErika López-ArribillagaRantao ZuoErika PalmieriRaphael ThuillierErin H. SeeleyRaphaela FritscheErnani PintoRaquel CumerasErnesto Martín-NúñezRaquel Fernandez-GarciaEsmat AliRashmi RashmiEsmat Farouk Ali AhmedRasmus Bovbjerg JensenEspen HansenRasoul MirzaeiEssam SolimanRathapon AsasutjaritEszter SzabadosRauf AbdurEugene NikolaevRaúl Díaz-MolinaEugenia BezirtzoglouRaúl González-DomínguezEugênio Gonçalves De AraújoRavichandra VemuriEugenio RagazziRavichandran RamasamyEun Jeong ParkRavinder GoyalEunha KimRaviraj ShindeEunice CarrilhoRaziye PourdarbaniEurydiki MichalisRebeca BustoEva BaranovičováRebecca J. WhelanÉva CsőszRegina Vincenzi OliveiraEva KudovaReiazul RehmanEva RamosReinhard ZelenyÉva SzabóRemington PoulinEva TvrdaRenata Novak KujundžićEva TvrdáRenata SoutoEvan PannkukRenato Barbosa FerrazEvelina Maria GosavRenyi LiuEvelyn OrsoRicardo Franco-DuarteEvelyne GozalRicardo M. BorgesEvert F.S. Van VelsenRicardo VallejoEwa Hanus-FajerskaRiccardo LeinardiEwa SzpyrkaRichard BroughtonEwa TomaszewskaRichard T. SayreEwelina HallmannRini Devijanti RidwanEylem Kulkoyluoglu CotulRita CasadonteEziuche Amadike UgboguRita PolitoF. Scott HallRituraj PurohitFabiana CrispoRobert AncuceanuFabiana Volpe-ZanuttoRobert BerkeczFabien ChevalierRobert Brawura-Biskupski-SamahaFabio Arturo IannottiRobert BrinsonFabio PaceRobert EckenstalerFabio SciubbaRobert GurkeFabrice DarcheRobert HäslerFadia YoussefRobert J. Van SaunFahad KhanRobert K. NaviauxFaisal RazaRobert KupczyńskiFaiyaz ShakeelRobert L. JarretFan KelongRobert N. NishimuraFang FangRobert T. MeansFangcong DongRoberta ZupoFarideh RaziRoberto BenelliFarshad DarvishiRoberto CannataroFarzaneh KordbachehRoberto ChiarelliFarzaneh SabbaghRoberto CiampagliaFatema BhinderwalaRoberto CodellaFatiha NassirRoberto ScicaliFatima O. SmagulovaRobin A. WilsonFatimah KhalafRobin WilsonFausto MaffiniRobson De FariasFederica ChiapporiRocío ChecaFederica De CastroRodrigo A. ContrerasFederica FogacciRodrigo CurvelloFederica MaggiRodrigo ValenzuelaFederico InfascelliRodrigo Vargas VitoriaFederico IovinoRodrigo ZaccaFederico Manuel GiorgiRohit MaharFederico Maria RubinoRohitash JamwalFederico TortaRoland TengolicsFei HeRomain DuvalFei PengRoman HusnikFelicite Noubissi-KamdemRoman K. PuzanskyFelipe José AidarRoman PuzanskiyFelix MaurerRoman SmolarczykFengjie SunRomeo T. CristinaFerdinando PaternostroRon KedemFerenc DomokiRonald B. BrownFerman ChavezRonald B. MooreFernando CardonaRongjin SunFernando Lopitz OtsoaRonit SionovFernando ReyesRosa María Giráldez-PérezFilippe Falcao-TebasRosa Maria N. MarcussoFiona A. HagenbeekRosa Maria Tapia-HaroFiorenzo LupiRosa PascaleFiroz AkhterRosa SuadesFlávia Giolo De CarvalhoRosaria VarìFleming GudaguntiRosario MareFlorin FilipRosilda Mara MussuryFodor MariettaRossana Rodríguez-CanulFrancesca AccioniRossella CaneseFrancesca CianiRoxana Liana LucaciuFrancesca GrazianoRoxana LucaciuFrancesca LatinoRubén Gil-SolsonaFrancesca MottarliniRuchika BhawalFrancesca ReggianiRueyleng LooFrancesca SilvagnoRui VitorinoFrancesca SistoRuitao YuFrancesca VasileRuitu LyuFrancesco AsciRuiyang BaoFrancesco AsnicarRundk HwaizFrancesco BalestriRune SlimestadFrancesco BiancoRuqiang XuFrancesco CacciolaRuth Hornedo-OrtegaFrancesco CanonicoRuwei LinFrancesco De ChiaraRuy A. LouzadaFrancesco InchingoloRuzilawati Abu BakarFrancesco Paolo FanizziRyan G. SnodgrassFrancesco SessaRyan S. PralleFrancisca RodriguesRyoma MichishitaFrancisco Cruz-SosaRyota HosomiFrancisco J. BarbaRyusuke NiwaFrancisco Javier Navas GonzálezS. M.Shakeel IqubalFrancisco Marlon Carneiro FeijóSaba GillFrancisco MesaSabina GaliniakFrancisco Rodrigues PintoSabino PachecoFrancisco SolanoSabrin IbrahimFranco CervellatiSabrina AnticoliFranco MutinelliSachin KumarFrancois GaaschtSaeed TarighiFrank C. KoSaeed YousefinejadFranko BurculSaichiro YokoyamaFrédéric DumurSaiful MalookFrederick M. LeMieuxSaima RubabFuhua HaoSajad WaniFujie YanSajal ShrivastavaFulvio RattoSakineh HajebrahimiFumikazu MatsuhisaSalam IbrahimFuming ZhangSalem ElkahouiGábor BaloghSalvatore BarrecaGábor SomlyaiSalvatore IaconoGabriel Del RioSameh ElhadyGabriela Alemán-EscondrillasSamo LešnikGaetano MarvertiSamson GuenneGaetano SantulliSamuel PitaGaia MeoniSamuel SilvestreGalia ZamaratskaiaSamuel X. ShiGalina NikolovaSandeep Kumar SinghGalya StanevaSándor GondaGang ChenSandor HosztafiGang LiSandra BarbalhoGary BurrSandra BodeauGaurav SharmaSandra GhelardoniGautam PareekSandra LealGema Frühbeck MartínezSandra RebeloGema Martinez-NavarreteSandro OrrùGen KanekoSang Gil LeeGengjun ChenSanja ZeljkovicGeoffrey C. KiteSanjin KovacevicGeorg BaslerSanket P. AnaokarGeorge A. KoumantakisSantiago Navarro LedesmaGeorge FthenakisSantosh A. MisalGeorge KokotosSantosh K. YadavGeorges MaestroniSantosh UpadhyayGeorgi As. GeorgievSan-Yuan ChenGeorgia-Eirini DeligiannidouSara Al-GhadbanGeorgios BlekasSara CasatiGeorgios PampalakisSara GranjaGeorgios PapadopoulosSara ZareiGeorgios TheodoridisSarah DuinGeorgios TsaniklidisSarah HannouGerald Fritz SchusserSarhang HamaGeraldine GueronSarkar M. Abe KawsarGerard Kian-Meng GohSarvesh Pratap KashyapGerard DumancasSaša ĐurovićGerardo Fernández BarberoSaša FrankGerardo Leyva-GómezSatoshi HagaGergő KallóSaulo Luz SilvaGergő TóthSaverio MuscoliGermán EbenspergerSavneet KaurGerson Aparecido Foratori-JuniorSawsan A. ZaitoneGert M. KostnerSean B. FainGhada Abd-Elmonsef MahmoudSebastian PiłsykGhader ManafiazarSebastian ZahnreichGhasem SolgiSebastien LacroixGhazala MustafaSeh-Hoon OhGheorghe IliaSeigo SanohGholamreza GohariSenthilkumar PalanisamyGiacomo LuciSeong Beom ChoGiampiero CaiSergi MaicasGianfranco PiconeSeveriano R. SilvaGianluca RizzoSéverine BärGina MendezSeverino PersechinoGinno MillanSeyed Hossein HoseinifarGino SeravalleShaaban ElnesrGiorgia ContaShadma W. AbdulwahabGiorgia PertileShagufta PerveenGiorgio SalutiShahab UddinGiorgio SmaldoneShahenda MahgoubGiorgio TregliaShaimaa SelimGiovanna BaronShamir CassimGiovanna RomanoShams TabrezGiovanna TrainaShane AustinGiovanna TranfoShangru LyuGiovanni BocciaShankarappa SridharaGiovanni Nicolao BertaShaohua WangGiovanni TarantinoShaoning JiangGiovanni VitaleSharad PurohitGirish Kumar GuptaShayne MasonGirish PattappaShehwaz AnwarGiulia BernardiniSheila SpadaGiulia FerrarazzoShenghao LiuGiulia PignataroShengru WuGiuliano RamadoriShenjin LvGiulio CeolottoSherif KaramGiuseppe AmorusoSherin SaheeraGiuseppe AstaritaShi YaoGiuseppe BiaginiShiang-Jong TzengGiuseppe LucarelliShigehiro KarashimaGiuseppe ManninoShigeho TanakaGiuseppe MartanoShigenobu ShibataGiuseppe MartelliShih-Yi HuangGiuseppe MurdacaShijie HeGiuseppe PagliaShimizu NaotoGiuseppe PiccioneShing-Hwa LiuGiuseppe SiragoShin-ichi KayanoGiustino OrlandoShivanand M. PudakalakattiGladys Oluyemisi Latunde-DadaShivankar AgrawalGleyson Kleber Do Amaral-SilvaShivraj YabajiGloria H. Y. LiShosuke ItoGonçalo J. JustinoShoulong DengGoran MitulovicShozo TomonagaGotthold GäbelShravan GiradaGraça SoveralShubai LiuGrace FarhatShugo MizunoGrazia GalleriShuichi SetoguchiGrażyna BudrynSigita JurkonienėGrażyna Jarząbek-BieleckaSihui MaGrażyna NowickaSilvano DragonieriGrażyna Odrowaz-SypniewskaSilvia Alejandra García-GascaGregorio PeronSilvia AziriovaGrzegorz BartoszSilvia CapannaGrzegorz GrześkSilvia FilippiGrzegorz ŁysiakSilvia LakatošováGrzegorz NowickiSilvia LandiGuadalupe Virginia Nevárez-MoorillónSilvia LiscianiGuan WangSilvia PampanaGuanbin SongSílvia PetronilhoGuanhui WuSilvia RocchiccioliGuan-Jhong HuangSilvia SvegliatiGuannan WangSimon CobboldGuillermo QuintásSimon Vlad LucaGulcin YildizSimona DanieleGünter MüllerSimona ErricoGuobin XiaSimona SaponaraGuofang ShenSimona TodiscoGuoxun ChenSimona ZaamiGurpreet Kaur AroraSimone CarottiGustavo EgeaSimone CarradoriGustavo Gonzalez-NevesSimone PernaGustavo PimentelŠimons ŠvirskisGustavo RosaniaSiqi HuGustavo Vieira De OliveiraSiranjeevi NagarajGyörgy SiposSiva PandaHabib RehmanSivapong SungpraditHadith RastadSiwei ZhangHafiz RizwanSlađan PavlovićHafiz-Muhammad-Umer FarooqiSlavica KvolikHai Long WangSlawomir GonkowskiHaiming LuoSmaoui SlimHaishu LinSmarak BandyopadhyayHajo HaaseSnehal JadhavHakan BozdoğanSnježana Mardešić-BrakusHamada ElwanSobhi M. GomhaHamdoon MohammedSohail QaziHamdy AbdelkaderSohini ChakrabortyHamid R. EghbalniaSoibam Khogen SinghHamidreza ArdalaniSolmaz SaremnezhadHammad AlamSongbo WangHanefí ÖzbekSoni DhruvKumarHang HuSonia Escandón-RiveraHang Soo ParkSonia FantoneHani Nasser AbdelhamidSonja GamsjaegerHanna JulienneSophie Antoine-JonvilleHanna KmitaSotirios SotiriouHanna MojskaSoumaya KouidhiHanne Christine BertramSoumyadev SarkarHanqing ChenSousuke ImamuraHany Ezzat KhalilSowjanya ThatikondaHany G. Abd El-GawadSpyridon KanellakisHany Hamdy ArabSpyros PapageorgiouHany I. SakrSree V. ChintapalliHao WuStafford VigorsHarada KoujiStamatina KallithrakaHarald C. KöfelerStanislav KotlyarovHardeep Singh TuliStanislaw DejaHassan El-RamadyStanisław KaliszHassan Y. EbrahimStanisław NiemczykHeather Jane WalkerStanisław WitkowskiHeba SahyonStankevičius VaidotasHector Gallart AyalaStanley ChanHector Garcia MartinStefan BroerHeiko BrennenstuhlStefan GaździńskiHenk KoppelaarStefan KranzHenrique P. NeivaStefan ZielenHerbert Ryan MariniStefania GalimbertiHermona SoreqStefania HanauHesham El-SeediStefania KinspergherHideyuki KawauchiStefania MitolaHimani PuniaStefania NoermanHirofumi FuruitaStefano CagninHirokazu FukuiStefano MenichettiHiromasa HasegawaStefano TizianiHiromitsu NegoroStefanos StefanouHironori YoshinoSteffen NeumannHiroshi KitagakiStephan WalchHiroshi MatsufujiStéphane BurteyHiroyuki KonnoStephanie DuguezHisaaki MiharaStephanie HingHoi Leong Xavier WongStergios BoussiosHong Sheng ChengSteve MooreHong-Duck KimSteven HeatonHongfei ZhaoStig MølstedHongwu MaStrzemski MaciejHongying (Hoy) ShenSubramanyam DasariHongying WangSudip PaulHoon KimSue-Joan ChangHorst Joachim SchirraSueli Coelho Da Silva CarneiroHosam El-AnsarySuisui HaoHoward A. YoungSukkum ChangHrvoje PavlovićSuling HuangHsiaowei LiaoSultan Ayesh Mohammed SaghirHsin-Yao WangSumio AkifusaHsin-Yu ChenSumit MukherjeeHsiuying WangSun Young ParkHsueh-Wei ChangSung Eun KimHuafeng WangSung Soo AhnHuali ZuoSung-ho LeeHuanwen ChenSung-Kun (Sean) KimHubert CharlesSupavit ChesdachaiHubert SytykiewiczSurendra RajpurohitHugo Martínez-RojanoSuresh NarayanasamyHuibin ZouSusan RazvanHui-Chao YanSusana B. BravoHung-Pin TuSusana FrancoHung-Tse HuangSusana P. PereiraHung-Yi ChuangSusana Sangiao-AlvarellosHye Suk LeeSusanne RospleszczHyeong-Geug KimSushant BangruHyung-Kyoon ChoiSushila SinghIan RossSusumu MuroyaIan WilsonSutthiwal SethaIbrahim Ahmed ShaikhSuvik AssawI-Chung LuSven F. GarbadeIgnacio Díez LópezSvend LindenbergIgor MorgunovSvetlana SarantsevaIgor PottosinSvetozár DluholuckýIk-Hwan HanSveva GrandeIldiko SzantoSwati JainIleana ConstantinescuSyed Azmal AliIlioara OnigaSyed Hani AbidiIlona GórnaSyed Haris OmarIlona JonuškienėSyed NabilImad KhanSyed Sarim ImamImmacolata Concetta TommasiSyed Sayeed AhmadIndumathi SathisaranSylvester Yao LokpoInes Bilic-ĆurčićSylwester ŚlusarczykIngeborg KlymiukSylwia OsowskaIngrid CipakovaSzabolcs BéniIngrid Rodríguez-BuenfilSzu-Chuan ShenInho YangT.P. KukinaInmaculada Ramírez-MacíasTabussam TufailIoanna ChatzigiannidouTadashi SanoIoanna NtaikouTadeusz AmbrozyIoannis IliasTadeusz KowalskiIoannis P. GerothanassisTae Hwan KimIoannis S. PaterasTaia Abd El-MageedIoannis ZabetakisTai-Young HurIoannis-Dimosthenis AdamakisTakashi KokudoIonela HoteaTakashi UebansoIrena Brčić KaračonjiTakashi YazawaIrene CampiTakayuki MasakiIrene PanderiTakehisa HanawaIreneusz SowaTakemichi FukasawaIrfan A. RatherTaku NakayamaIrina IelciuTakuji OhyamaIrma TchokhonelidzeTakuma InagawaIrmanida BatubaraTakuma MatsubaraIsabel AfonsoTakuya KoieIsabel Kinney Ferreira De Miranda SantosTalal A. ZariIsabel Ten-DoménechTamar BasishviliIsabella PigaTamara Lazarevic-PastiIsabella RussoTamás LetohaIsabella ZanellaTanja Grubić KezeleI-Shiang TzengTanja J. MiličićIshita ChatterjeeTanja M. HessIshwarlal JialalTao LiIsidro Vitoria MiñanaTao LinItamar GonçalvesTaras P. PasternakIuga MadalinaTariq AbdulKarim AlalwanIulia-Ioana Stanescu-SpinuTariq IsmailIva LakićTarique HussainIvan BratchenkoTarun Kumar DuaIvan Cruz-ChamorroTateaki NaitoIvan DimauroTatiana AstrelinaIvan M. SavicTatiana ChristidesIvan SavicTatiana V. ShelengaIvana GunjačaTatijana ZemunikIvana JarakTatjana KošmerlIvana NovakTatjana PirmanIvana ŠkrlecTatjana RadosavljevićIvana StanimirovaTatsuo KandaIvar LundTatsuya MorimotoIwona CiereszkoTatyana BaltinaIwona KonopkaTeodora Emilia ColdeaIwona KowalskaTeresa Ayora-TalaveraIwona Piatkowska-ChmielTeresa OlejniczakIwona ŚcibiszTeresa PerraIwona SzydłowskaTeruhiko ImamuraIzabela FeckaTeruo MiyazakiIzabela Szymczak-PajorTesfalem WelearegayJ. Michael SorrellTessa CamenzindJacek KominekTetsuya IkedaJacek SmerekaTetsuya MatsukawaJacob FolzTetsuya ShiuchiJacopo Junio Valerio BrancaThaís R. SilvaJacopo LucchettiTharun Teja PonduruJae-Ho LeeTheodora PapamitsouJae-Hyung ParkTheofanis VavilisJae-Won LeeThierry HocJagdish Kumar JaiswalThierry RabilloudJaime Bustos-MartínezThomas BartnikasJair PutzkeThomas CaspariJakub SzperlikThomas KöcherJalal TaneeraThomas PolakJalil Ghassemi NejadThomas WeichhartJames G. GrannemanTianfa XieJames La ClairTianhua NiuJames LeighTiantian ZhangJames McCullaghTijana SubotičkiJames P. BennettTilili BarhoumiJamioł-Milc DominikaTim WehnerJan BocianowskiTímea SzekerczésJan KorbeckiTimothy D. VeenstraJan PaczesnyTimothy HamerlyJan WinklerTina B. McKayJana MašlankováTing LiuJana Šic ŽlaburTirthadipa Pradhan-SunddJandeli NiemandTobias ReiffJane CoxTom L. BroderickJanez JazbecTomabu AdjobimeyJanis LiepinsTomáš AdamJanthima MethaneethornTomas BogielJarosław CzyżTomáš HavránekJaroslaw KierkusTomasz FlorowskiJason T. TzenTomasz FrączykJason T.C. TzenTomasz PowrózekJaswinder K. SethiTomasz SledzinskiJavad MottaghipishehTomasz W. KaminskiJaved IqbalTomasz WypychJavier ModregoTomasz ZielinskiJavier SaurinaTomislav MeštrovićJavier SeravalliTommaso LomonacoJawad KhanTomohiko KomatsuJay TrivediTonali Blanco AyalaJean-Claude DesfontisToncho DinevJędrzej AntosiewiczTong WangJeella Z. AcedoTonia VassilakouJeevan PrasainTony George JacobJelena DinicToon DuijnhouwerJelena LozoToshihiro Sakurai SakuraiJelena Marković FilipovićTossaporn SeeherunvongJelena VladićTracey L. WoodliefJennifer GubitosaTrevor NyakudyaJennifer MytychTsung-Han ChouJennifer S. ForbeyTsun-Thai ChaiJens HarderTsvetelina VelikovaJens JordanTuba Çiğdem OğuzoğluJeong-An GimTuba EsatbeyogluJeonghoon HaTudor Lucian PopJeremy EverettTuo MengJeremy KoelmelTusty-Juan HsiehJeremy Van RaamsdonkTuuli KaambreJerod J. HurstTzong-Yuan WuJérôme RobertUjjal Kumar BhawalJerzy AdamskiUlf LiebalJerzy JuśkiewiczUlisses Moreno-CelisJessica Costa-GudaUlrich DesselbergerJessica IorioUlrich GergsJessy EtienneUlrika Von DöbelnJesus BalsindeUma Maheswari Devi PalemaplliJesus LacalUma ShankerJesus Pujol SaludUroš GašićJesús Siquier CollUsama Ramadan AbdelmohsenJia-Feng ChangUte WölfleJiahua GuoV.G AbilashJiajia SongVadim YansholeJialing ZhangVaideesh ParasaramJian LiVaithinathan SelvarajuJian ZhangValentina BucciarelliJiangeng HuangValentyn OksenychJianguo XiaValeria RighiJianhong ChingValeria SülsenJianing MiValeria ToscanoJianjun WenValerie CopieJianzhou CuiValerija DunkićJianzhu LiuValerio LeoniJichun ZhaoValiollah PalangiJie ZhuVanesa EstebanJieun OhVanessa BrinkmannJifu LiVanessa RubioJin BaiVan-Giau VoJing ZhangVanja TadićJingen ZhuVasil AtanasovJinghua WangVasil N AtanasovJinhae KimVasileios PapatsirosJinsong ZhangVasily AleshinJinwei ZhangVassilios DotasJinyoung BarnabyVassya BankovaJitendra KanshanaVendula HepnarovaJitendra KumarVeronica GhiniJiyeon HamVeronika HuntošováJize XueVesa Cosmin MihaiJoan AlbiolVeysel TuranJoan Carles Escola-GilVíctor García-GonzálezJoana BarbosaVictor MartinJoana F. SacramentoVictoria L. StevensJoana GasparVictoriya A. GeorgiyantsJoana PintoVida JuozaitienėJoana RodriguesVijay HegdeJoanna GiebultowiczVijay MishraJoanna L. FoxVijaya Anand ArumugamJoanna Pajdak-CzausVijeta SharmaJoanna PodlasińskaVikas KannojiyaJoanna SmyczyńskaVikas KumarJoanna ZemłaVikash KumarJoanne K. KelleherVimal Kumar BalasubramanianJoão Henrique Ghilardi LagoVinay Kumar TyagiJoão M. MirandaVincenzo CarcangiuJoão Micael LeçaVincenzo MontinaroJoao Ramalho-SantosVincenzo ParrinoJoão Ramalho-SantosVincenzo TufarelliJoaquim CarrerasVinícius CantarelliJoaquim JaumotVinny NegiJochen D. SchipkeVinod K NarayanaJochen SchneiderVinod VermaJodeh ShehdehVito Giuseppe D'AgostinoJoe MillwardVitor ParolaJohann SchinnerlVittorio FineschiJohannes HoferVivek PecheJohannes MeiserViviana BarraJóhannes ReynissonVlad PădureanuJohannes WöstemeyerVladimir A. KorshunJohn B. VincentVladimir JurisicJohn Bertram VincentVladimir KalininJohn HendryVladimir SobolevJohn J. GuersVolodymyr PaukJohn M. PerryWafaa A. Abd El-GhanyJohn MorganWai San CheangJohn O. OnukwuforWalaa NegmJohn QuiñonesWalther BildJohn W. SteeleWanda MączkaJoias HammanWang LeiJoice Tom JobsWarley BorgesJolanta JaworekWaseem El-HuneidiJonathan FusiWayne CarterJong Hwa LeeWayne CarverJoonhoon KimWayne Thomas ShierJoon-hoon KimWei JiaJoo-Youn ChoWei ZhangJordi Capellades TomàsWei ZhongJorge A. LópezWei-Hsiang ChangJorge AntunesWeisi LiuJorge Gutiérrez-CuevasWeiwei CuiJorge Hernández-VaraWen Chieh LiaoJorge Limon-RomeroWen ShiJorge R. Miranda-MassariWenpeng ZhangJoris BeldWenting GuoJos HagemanWen-Wei Robert HsuJosé A. LupiáñezWenxin SongJosé A. MonrealWenxin TongJosé Antonio RodríguezWesley Lyeverton Correia RibeiroJosé Carlos Tavares CarvalhoWidiastuti SetyaningsihJosé Cleiton Sousa Dos SantosWiesław BielasJosé Enrique Moral-GarcíaWilco ZijlmansJosé Guadalupe Herrera HaroWilhelm BlochJosé Luis Pérez-CastrillónWilliam Bryan TerzaghiJosé Luiz De Brito AlvesWilliam PotterJose Manuel MoralesWilliam T. BeckJosé Pedraza ChaverriWilman CarrilloJosé Pedro AndradeWioletta PawlukowskaJosé Pedro Cerón-CarrascoWiramon RungratanawanichJosé Sousa CâmaraWissem MnifJosef FinstererWladimir Bocca Vieira De Rezende PintoJosef KameníkWoei Yenn TongJosefa Gonzalez-SantosWofram GronwaldJosefina CasasWojciech JelskiJosely KouryWojciech Jerzy PietrońJosep CentellesWojciech ŁuczajJosep JulveWojciech SzlasaJoseph P. DewulfWolfgang BuckelJosé-Tomás NavarroWolfgang SattlerJoshua D. ChandlerWong Yong FooJosselin LupetteWoon-Man KungJuan Antonio Rendón HuertaXavier De La TorreJuan Francisco CabelloXavier Gallart-PalauJuan Gerardo Reyes-GarcíaXian LuoJuan José Martínez NicolasXiang LiJuan Manuel Guzmán-FloresXiang WangJuan Monribot-VillanuevaXiangfeng KongJuan Mozas-MorenoXiangming JiJuan Pablo NicolaXianjiang LiJuan PeragónXiao XuJuan TorrasXiaobo HeJuana ChagasXiaofeng LiuJuei-Tang ChengXiaojie LiuJuergen HahnXiao-Jing WangJulia M. RosaXiaolong YangJulia NowowiejskaXiaorong FuJulián AragonésXiaowei LuoJulian FriebelXiaoyan DuJulián Rodriguez TalouXiaoyang SuJulian SwierczynskiXiaoyu LiaoJuliana Velasco De Castro OliveiraXin ChenJulien BoccardXingyou GuJulio AlarconXinheng YuJulita KulbackaXinigang Li‪Junia Nogueira De BritoXinxing WangJunrui ChengXiuqin YangJurga BudieneXóchitl TrujilloJürgen ZanghelliniXuhu GuoJussara AmatoXuhuiqun ZhangJustyna HermanowiczXun-Bo ZhouJyothi F NagajyothiYahia Z. HamadaK. GulyaYan LamK. SakthisudhanYan Wang (China)K.B. HebbarYan Wang (Pakistan)Ka-hei Kenneth LoYaohua YangKai CaiYaoxiang LiKai ZhangYasser KandilKalevi KairemoYasuka MatsunagaKalle KilkYasuko FujitaKanwal RehmanYasuko TanakaKaoru YoshidaYasunari MatsuzakaKarel D. CapekYasunori YaoitaKaren A. KopciukYasushi ShibataKaren LindsayYasuyuki FukuharaKarl BurgessYen-Chou KuanKarl Henrik GrinnemoYen-Lin LiuKarol WróblewskiYeongho KimKarolina Anna Wojtunik-KuleszaYhiya AmenKarolina BarcauskaiteYi WangKarolina ChilickaYi-Hsien HsiehKarolina GołąbekYijun PanKarolina HoffmannYimei JiaKarolina M. StepienYing YanKarolina Wojtunik-KuleszaYing YangKaroly HebergerYing-Chieh ChenKarthick RajanYingfeng ZhengKashmir SinghYi-Sub KwakKaspar TootsiYi-Ting LinKatarína MarákováYiwei MaKatarina SmiljanićYiwen ZhangKatarzyna BergmannYngve StenstrømKatarzyna CzyżYohannes W. WoldeamanuelKatarzyna KaczyńskaYohei ShirakamiKatarzyna Komosinska-VassevYoko SatoKatarzyna Kosicka-NoworzyńYona KeisariKatarzyna OszajcaYongbo XueKatarzyna PawlakYonghe MaKatarzyna Winsz-SzczotkaYonghong ZhuKatarzyna ŻarczyńskaYonghuan YunKatarzyna ZorenaYongjian LiKaterina KotzampassiYong-Moon LeeKateryna LystvanYongqiang LiuKatherine N. ThekenYongsoo ChoiKathleen A.R. SchildrothYongsoo KimKatja Dettmer-WildeYongxiang SongKatsuki TsuchiyamaYongzhen LiuKatyayani TatipartiYoonJung LeeKavita SharmaYoshifumi SaishoKazi RafiqYoshiharu FujiiKazuhiro P. IzawaYoshihiro FukumotoKazuo KobayashiYoshihiro ShidojiKazutaka HirakawaYoshihiro ToyaKees NoordamYoshinari UeharaKefeng LiYoshinobu OdakaKei MaruyamaYoshinori OkamotoKeiko HosohataYoshiro SaitoKeith RochfortYoshiya OdaKen-ichiro MinatoYoshiyuki NakamuraKenji HayashidaYounès DelleroKenji IkeharaYouness LimamiKenneth E. OverturfYoung-Min ParkKensuke KumeYoung-Sang KimKesawat Mahipal SinghYoussef RouphaelKe-Vin ChangYouzhong LiuKevin FitzsimmonsYu KoyamaKevin LamoteYu SunKevork PeltekianYuan GaoKeyvin DarneyYuan XuKhaled M. DarwishYuan-Bin ChengKhemayanto HidayatYuanmei ZuoKhizar HayatYuanyi FengKhursheda ParvinYuanyuan ShiKhurshid AhmadYu-Chieh ChenKihwan ChoiYuemin BianKi-Kwang OhYuesheng DongKim HellgrenYu-Fang HuangKim KultimaYuh-Chiang ShenKinga MusiałYuichi TsuchiyaKira V. VyatkinaYuji ShimizuKishneth PalanivelooYujue WangKishu RanjanYuko HaradaKit Leong CheongYulian VoynikovKlaus-Dieter SchlüterYuliana Nancy DewiKo FujimoriYuliya I. RaginoKoichi NakazatoYumin DaiKomuraiah MyakalaYun HuangKonrad Kamil HozyaszYun ShiKonrad SzychowskiYunlei LiKonstantinos GardikisYun-Ping LimKonstantinos KouremenosYunping QiuKoraljka Gall TroseljYunsong LiuKorawan SringarmYusof KamisahKornanong YuenyongchaiwatYusuf SertKosuke SaitoYuta TanizakiKotaro MatsumotoYuyin ZhouKoteswara Yerra RaoZachary S. ClaytonKoumantakis GeorgeZachary T GraberKourosh HooshmandZahra TalebiKoutavarapu RavindranadhŻaneta Kimber-TrojnarKrishnamoorthy SrikanthZbigniew J. WitczakKristaps KlavinsZbigniew RaszewskiKristian PetersZeeshan HamidKristina EndresŽeljko DolijanovićKristine GriffettŽeljko JakšićKrystyna PawlasZenon CzubaKrzysztof GorynskiZhan ShuKu Youn BaikZhaohai QinKuang-Tai KuoZhenglin YangKuan-Han LeeZhengxiang HuangKuen-Cheh YangZhenwen ZhaoKuldeep AttriZhibin LiuKumail K. MotianiZhifeng DuKumar Chandan SrivastavaZhihao ChenKumar KatraguntaZhijun WangKunihisa KobayashiZhi-min ZhangKunka Mohanram RamkumarZhiping LiuKun-Ling TsaiZhiyong HuKun-yun YehZhiyuan MengKushagra BansalZhongbing LuKyle BurghardtZhonggang FengKyle McLeanZijian ZhangKylie D. RockZivile UseckaiteKyu Young ChoiZixin ChenKyung Pyo KangZizy Ibrahim ElbialyKyung-Jin MinZon Weng LaiKyung-Min LimZongxiang TangKyuseok KimZoran MinicLaila ZikoZorica Kauric-KleinLamine Baba-MoussaZoya MarinovaLarissa BuedenbenderZrinka KovarikLars LinsenZsolt GállLars-Olof BjörnZsolt SzűcsLassaâd BelbahriZulfiqarali G AbbasLasse LindahlZunyao WangLaszlo TretterZuyi (Jacky) HuangLaura Aracely Contreras-AnguloZygfryd Witkiewicz

